# Supplementary material for: Conspiracy beliefs and negative attitudes towards outgroups in times of crises: Experimental evidence from Germany
Source: PLoS One. 2024 Nov 20;19(11):e0312418. doi: 10.1371/journal.pone.0312418 (PMC11578531; doi:10.1371/journal.pone.0312418)
Supplement: S1 File — (DOCX) [file pone.0312418.s001.docx]

# Supporting Information

## Summary statistics

Table S1: Distribution of the responses on the likert scale for the conspiracy beliefs question across treatment groups

| Items | **Diseases** | | **Economy** | | **Wars** | |
| --- | --- | --- | --- | --- | --- | --- |
| 1, do not believe at all | 178 | 34.1% | 78 | 14.8% | 15 | 3.1% |
| 2 | 127 | 24.3% | 102 | 19.4% | 49 | 10.0% |
| 3 | 80 | 15.3% | 127 | 24.1% | 71 | 14.5% |
| 4 | 73 | 14.0% | 127 | 24.1% | 151 | 30.8% |
| 5 | 34 | 6.5% | 65 | 12.4% | 124 | 25.3% |
| 6, completely believe | 30 | 5.7% | 27 | 5.1% | 80 | 16.3% |

Table S2: Summary statistics of all variables

| **Variable** | **N = 1,972** |
| --- | --- |
| Treatment group |  |
| Control | 488 (25%) |
| Diseases | 503 (26%) |
| Economy | 505 (26%) |
| Wars | 476 (24%) |
| Belief in conspiracy |  |
| 1 | 248 (18%) |
| 2 | 260 (18%) |
| 3 | 253 (18%) |
| 4 | 329 (23%) |
| 5 | 200 (14%) |
| 6 | 125 (8.8%) |
| NA | 557 |
| Male | 953 (48%) |
| Education |  |
| Primary education/None | 315 (16%) |
| Secondary education | 554 (28%) |
| Abitur | 1,103 (56%) |
| Religion |  |
| Christian | 1,111 (56%) |
| Muslim | 76 (3.9%) |
| Other | 36 (1.8%) |
| None | 749 (38%) |
| Migration background | 507 (26%) |
| Survey administration |  |
| Online | 550 (28%) |
| Paper-and-pencil | 1,422 (72%) |
| East Germany | 427 (22%) |
| **Variable (table continued)** | **N = 1,972** |
| Left-right scale |  |
| 1 (left) | 73 (3.8%) |
| 2 | 121 (6.4%) |
| 3 | 350 (18%) |
| 4 | 292 (15%) |
| 5 | 575 (30%) |
| 6 | 264 (14%) |
| 7 | 126 (6.6%) |
| 8 | 76 (4.0%) |
| 9 | 12 (0.6%) |
| 10 (right) | 16 (0.8%) |
| NA | 67 |
| Political party preference |  |
| CDU | 362 (19%) |
| SPD | 213 (11%) |
| Greens | 471 (24%) |
| FDP | 126 (6.5%) |
| The Left | 97 (5.0%) |
| AfD | 68 (3.5%) |
| Other | 48 (2.5%) |
| Undecided | 472 (24%) |
| Ineligible | 92 (4.7%) |
| Missing | 23 |
| Political party preference  (excluding undecided and  ineligible voters) |  |
| CDU | 362 (26%) |
| SPD | 213 (15%) |
| Greens | 471 (34%) |
| FDP | 126 (9.0%) |
| The Left | 97 (6.9%) |
| AfD | 68 (4.8%) |
| Other | 68 (4.8%) |
| Missing | 567 |

## Balance checks for randomized groups

Figure S1: T-Test results for a range of demographic variables by treatment groups


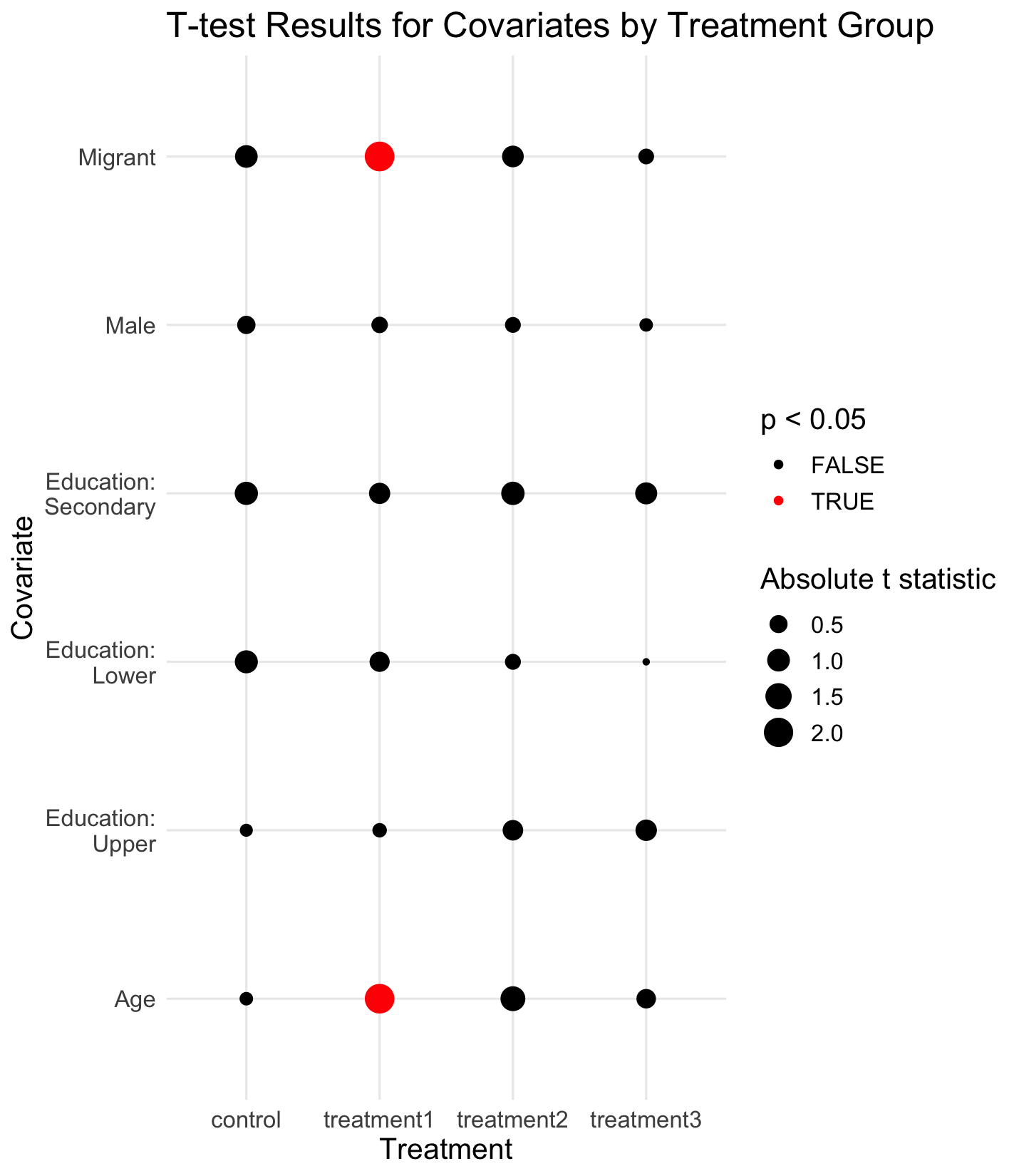


## Results of the regression analyses

Table S3: Effect of exposing respondents to a conspiracy-oriented framing of crises on negative attitudes towards outgroups (without control variables)

|  | *Dependent variable:* | | | | | | |
| --- | --- | --- | --- | --- | --- | --- | --- |
|  | Average index | US-Americans | Chinese | Russians | Jews | Refugees | Muslims |
|  | (1) | (2) | (3) | (4) | (5) | (6) | (7) |
| Treatment |  |  |  |  |  |  |  |
| (ref.: Control Gr.) |  |  |  |  |  |  |  |
| Disease | 0.37^**^ | 0.35^*^ | 0.47^**^ | 0.35^*^ | 0.41^**^ | 0.26 | 0.50^**^ |
|  | (0.13) | (0.15) | (0.17) | (0.16) | (0.15) | (0.16) | (0.18) |
| Economy | 0.39^**^ | 0.36^*^ | 0.58^***^ | 0.55^***^ | 0.21 | 0.27 | 0.45^*^ |
|  | (0.13) | (0.15) | (0.17) | (0.16) | (0.15) | (0.16) | (0.17) |
| Wars | 0.44^***^ | 0.42^**^ | 0.66^***^ | 0.52^**^ | 0.34^*^ | 0.26 | 0.65^***^ |
|  | (0.13) | (0.15) | (0.17) | (0.16) | (0.15) | (0.17) | (0.18) |
| Observations | 2,045 | 2,045 | 2,045 | 2,045 | 2,045 | 2,045 | 1,944 |
| Adjusted R^2^ | 0.01 | 0.00 | 0.01 | 0.01 | 0.00 | 0.00 | 0.01 |

*Note*: The Table shows OLS regression coefficients and standard errors (in parentheses). To ensure that respondents did not rate their ingroup, we removed Muslim respondents for the Muslim ratings. The total number of observations differs from the number of observations (n = 1,972) in the main models presented in the manuscript (Table 2), as those models include two control variables, and cases with missing values for these control variables were removed from the analysis. *p<0.05; **p<0.01; ***p<0.001

Table S4: Effect of exposing respondents to a conspiracy-oriented framing of crises on negative attitudes towards outgroups (with additional control variables)

|  | *Dependent variable:* | | | | | | |
| --- | --- | --- | --- | --- | --- | --- | --- |
|  | Average index | US-Americans | Chinese | Russians | Jews | Refugees | Muslims |
|  | (1) | (2) | (3) | (4) | (5) | (6) | (7) |
| Treatment |  |  |  |  |  |  |  |
| (ref.: Control Group) |  |  |  |  |  |  |  |
| Disease | 0.40^**^ (0.13) | 0.39^*^ (0.15) | 0.51^**^ (0.16) | 0.42^**^ (0.16) | 0.43^**^ (0.15) | 0.28 (0.15) | 0.46^**^ (0.17) |
| Economy | 0.37^**^ (0.13) | 0.36^*^ (0.15) | 0.55^***^ (0.16) | 0.52^**^ (0.16) | 0.18 (0.15) | 0.24 (0.15) | 0.38^*^ (0.17) |
| Wars | 0.43^***^ (0.13) | 0.42^**^ (0.16) | 0.68^***^ (0.17) | 0.50^**^ (0.16) | 0.31^*^ (0.15) | 0.24 (0.16) | 0.60^***^ (0.17) |
| Age | 0.02^***^ (0.00) | 0.01^**^ (0.00) | 0.03^***^ (0.00) | 0.03^***^ (0.00) | 0.01^**^ (0.00) | 0.01^**^ (0.00) | 0.03^***^ (0.00) |
| Male | 0.36^***^ (0.09) | 0.26^*^ (0.11) | 0.39^**^ (0.12) | 0.32^**^ (0.12) | 0.47^***^ (0.11) | 0.35^**^ (0.11) | 0.36^**^ (0.12) |
| Education |  |  |  |  |  |  |  |
| (ref.: Primary) |  |  |  |  |  |  |  |
| Secondary education | -0.31^*^ (0.14) | -0.12 (0.18) | -0.36 (0.19) | -0.17 (0.18) | -0.65^***^ (0.17) | -0.23 (0.18) | -0.09 (0.19) |
| *Abitur* | -0.60^***^ (0.14) | -0.36^*^ (0.17) | -0.51^**^ (0.18) | -0.44^*^ (0.18) | -0.86^***^ (0.16) | -0.80^***^ (0.17) | -0.48^**^ (0.18) |
| Religion |  |  |  |  |  |  |  |
| (ref.: Christian) |  |  |  |  |  |  |  |
| Muslim | 0.24 (0.25) | 0.56 (0.31) | 0.36 (0.33) | -0.04 (0.33) | 0.80^**^ (0.30) | -0.50 (0.31) |  |
| Other | 0.06 (0.35) | 0.29 (0.42) | -0.25 (0.46) | -0.07 (0.44) | 0.22 (0.41) | 0.11 (0.43) | 0.18 (0.45) |
| None | 0.31^**^ (0.10) | 0.51^***^ (0.12) | 0.10 (0.13) | 0.24 (0.13) | 0.40^***^ (0.12) | 0.27^*^ (0.12) | 0.09 (0.13) |
| Migrant background | 0.10 (0.11) | 0.27^*^ (0.13) | -0.04 (0.14) | -0.17 (0.14) | 0.23 (0.13) | 0.20 (0.13) | 0.27 (0.14) |
| Paper-and-Pencil | 0.20 (0.11) | 0.11 (0.13) | 0.21 (0.14) | 0.31^*^ (0.14) | 0.16 (0.12) | 0.20 (0.13) | 0.20 (0.14) |
| East Germany | -0.11 (0.12) | -0.07 (0.14) | -0.29 (0.15) | -0.51^***^ (0.15) | 0.20 (0.14) | 0.12 (0.14) | 0.08 (0.15) |
| Party preference |  |  |  |  |  |  |  |
| (ref.: CDU/CSU) |  |  |  |  |  |  |  |
| SPD | -0.28 (0.17) | 0.23 (0.21) | -0.40 (0.22) | -0.45^*^ (0.22) | -0.22 (0.20) | -0.57^**^ (0.21) | -0.47^*^ (0.23) |
| Greens | -0.49^***^ (0.14) | 0.12 (0.17) | -0.51^**^ (0.18) | -0.53^**^ (0.18) | -0.49^**^ (0.16) | -1.03^***^ (0.17) | -0.99^***^ (0.19) |
| FDP | -0.35 (0.20) | -0.38 (0.25) | -0.33 (0.27) | -0.59^*^ (0.26) | -0.43 (0.24) | -0.04 (0.25) | -0.20 (0.27) |
| The Left | -0.30 (0.23) | 0.67^*^ (0.28) | -0.24 (0.30) | -0.87^**^ (0.29) | -0.10 (0.27) | -0.96^***^ (0.28) | -0.51 (0.31) |
| AfD | 0.61^*^ (0.26) | 0.09 (0.32) | 0.21 (0.34) | -0.43 (0.33) | 0.36 (0.30) | 2.81^***^ (0.32) | 2.06^***^ (0.34) |
| Other | -0.05 (0.30) | 0.02 (0.37) | 0.41 (0.40) | -0.19 (0.39) | -0.26 (0.35) | -0.21 (0.37) | 0.31 (0.40) |
| Undecided | 0.08 (0.14) | 0.42^*^ (0.17) | -0.22 (0.18) | -0.13 (0.18) | 0.05 (0.16) | 0.28 (0.17) | 0.08 (0.18) |
| Ineligible | 0.64^**^ (0.23) | 0.70^*^ (0.28) | 0.18 (0.31) | 0.27 (0.30) | 0.73^**^ (0.27) | 1.33^***^ (0.29) | 1.31^***^ (0.32) |
| Observations | 1,949 | 1,949 | 1,949 | 1,949 | 1,949 | 1,949 | 1,877 |
| Adjusted R^2^ | 0.09 | 0.03 | 0.08 | 0.08 | 0.07 | 0.15 | 0.14 |

*Note*: The Table shows OLS regression coefficients and standard errors (in parentheses). To ensure that respondents did not rate their ingroup, we removed Muslim respondents for the Muslim ratings. The total number of observations differs from the number of observations (n = 1,972) in the main models presented in the manuscript (Table 2), since the models presented here include additional control variables, and cases with missing values for these control variables were removed from the analysis. *p<0.05; **p<0.01; ***p<0.001

Table S5 - Interaction effect of exposing respondents to a conspiracy-oriented framing of crises and respondents’ conspiracy belief inclinations on negative attitudes toward outgroups

|  | *Dependent variable:* | | | | | |
| --- | --- | --- | --- | --- | --- | --- |
|  | Average index | US-Americans | Chinese | Russians | Jews | Refugees |
|  | (1) | (2) | (3) | (4) | (5) | (6) |
| Treatment (ref.: Disease) |  |  |  |  |  |  |
| Economy | 0.37 | 0.04 | 0.56 | 0.51 | 0.11 | 0.67 |
|  | (0.29) | (0.35) | (0.37) | (0.37) | (0.34) | (0.37) |
| Wars | -0.14 | -0.73 | -0.03 | -0.25 | -0.20 | 0.53 |
|  | (0.35) | (0.43) | (0.46) | (0.46) | (0.42) | (0.46) |
| Conspiracy beliefs | 0.31^***^ | 0.21^**^ | 0.25^**^ | 0.23^**^ | 0.31^***^ | 0.57^***^ |
|  | (0.06) | (0.07) | (0.08) | (0.08) | (0.07) | (0.08) |
| Age | 0.02^***^ | 0.01^**^ | 0.04^***^ | 0.03^***^ | 0.02^***^ | 0.02^***^ |
|  | (0.00) | (0.00) | (0.00) | (0.00) | (0.00) | (0.00) |
| Migration background | 0.09 | 0.24 | -0.01 | -0.12 | 0.22 | 0.09 |
|  | (0.12) | (0.15) | (0.16) | (0.16) | (0.15) | (0.16) |
| Economy x Conspiracy beliefs | -0.18^*^ | -0.06 | -0.20 | -0.16 | -0.16 | -0.32^**^ |
|  | (0.09) | (0.11) | (0.12) | (0.11) | (0.11) | (0.11) |
| Wars x Conspiracy beliefs | -0.06 | 0.12 | -0.05 | 0.01 | -0.07 | -0.33^**^ |
|  | (0.09) | (0.11) | (0.12) | (0.12) | (0.11) | (0.12) |
| Observations | 1,415 | 1,415 | 1,415 | 1,415 | 1,385 | 1,415 |
| Adjusted R^2^ | 0.07 | 0.02 | 0.07 | 0.06 | 0.03 | 0.06 |

Note: The Table shows coefficients and standard errors of the main OLS regression of the indicated outcome on the disease, economy, and wars treatment dummies and incorporating an interaction term of the treatment conditions and the conspiracy beliefs while controlling for age and migration background. *p<0.05; **p<0.01; ***p<0.001

## Factor Analysis Results

We performed a factor analysis focusing on our six outcome variables: American, Jewish, Chinese, Russian, Refugees, and Muslims. We used principal component factors, and one factor was retained for analysis. We applied an oblique promax rotation. The total variance explained by the single factor is approximately 65.04%. The loadings on this factor indicate a strong relationship between each variable and the factor, with loadings ranging from 0.7379 to 0.8460. The uniqueness values, representing the variance in each variable not explained by the factor, are relatively moderate, suggesting that a significant portion of each variable's variance is accounted for by the factor. A likelihood ratio test suggests that the factor model is a much better fit than one assuming no relationship among the variables.

Method: Principal-Component Factors

Retained Factors: 1

Rotation: Oblique Promax (Kaiser Off)

Number of Parameters: 6

Variance and Proportion

Total Variance Explained by Factor 1: 3.90240

Proportion of Variance Explained: 65.04%

Table S6 - Rotated factor loadings (pattern matrix) and unique variances

| Variable | Factor1 | Uniqueness |
| --- | --- | --- |
| American | 0.7379 | 0.4555 |
| Jew | 0.7997 | 0.3604 |
| Chinese | 0.8351 | 0.3026 |
| Russian | 0.8172 | 0.3322 |
| Refugee | 0.7984 | 0.3626 |
| Muslims | 0.8460 | 0.2843 |

LR test: independent vs. saturated: chi2(10) = 7318.54 Prob>chi2 = 0.0000

Appendix Figure S2: Correlation matrix of outcome variables


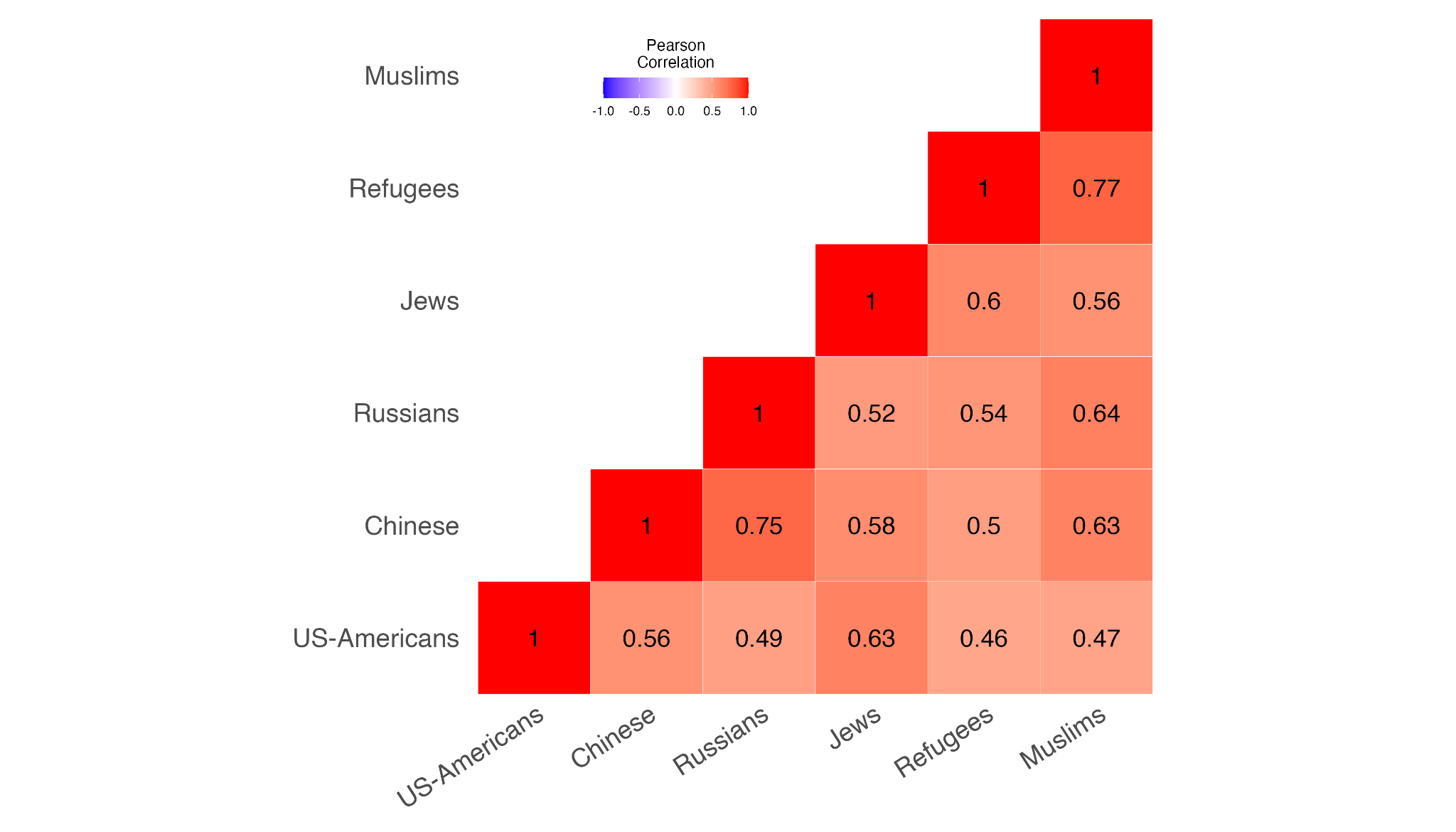


The correlation matrix above indicates that our outcome variables are highly correlated with the exception of US-Americans and Russians, Refugees, and Muslims, which are moderately correlated.

## Heterogenous treatment effects across political ideology

Appendix Table S7 - Effects of treatment conditions on average outgroup index conditional on ideology

|  | *Dependent variable:* |
| --- | --- |
|  | Average index |
| Treatment (ref.: Control group) |  |
| Diseases | 0.79^***^ (0.19) |
| Economy | 0.49^*^ (0.19) |
| Wars | 0.53^**^ (0.19) |
| Ideology (ref.: Left) |  |
| Center | 0.90^***^ (0.19) |
| Right | 1.11^***^ (0.30) |
| Age | 0.02^***^ (0.00) |
| Migration background | 0.13 (0.10) |
| Diseases x Center | -0.82^**^ (0.27) |
| Economy x Center | -0.54^*^ (0.27) |
| Wars x Center | -0.29 (0.27) |
| Diseases x Right | -0.32 (0.41) |
| Economy x Right | 0.73 (0.42) |
| Wars x Right | -0.03 (0.41) |
| Observations | 1,905 |
| Adjusted R^2^ | 0.08 |

Note: The Table shows coefficients and standard errors of the main OLS regression of the indicated outcome on the disease, economy, and wars treatment dummies and incorporating an interaction term of the treatment conditions and the ideology variable while controlling for age and migration background. *p<0.05; **p<0.01; ***p<0.001
